# Parameter Optimization of Laser Direct-Write Patterning on Indium Tin Oxide/Polycarbonate Thin Films Using Multi-Performance Characteristics Analysis

**DOI:** 10.3390/ma14195808

**Published:** 2021-10-04

**Authors:** Yi-Cheng Chen, Yi-Kai Hsiung, Chih-Yuan Chang, Shih-Fu Ou

**Affiliations:** Department of Mold and Die Engineering, National Kaohsiung University of Science and Technology, Kaohsiung 807618, Taiwan; smn874022000@gmail.com (Y.-C.C.); zxc75231@gmail.com (Y.-K.H.)

**Keywords:** laser direct-write, ITO thin film, Taguchi methods, analysis of variance

## Abstract

Indium tin oxide (ITO) thin films on polycarbonate (PC) substrates were patterned using the laser direct-write (LDW) technique to form an isolation line. The effect of the LDW parameters (power, pulse repetition rate, and defocusing distance) on the isolation line width, depth and roughness of the PC within the line was investigated. Additionally, the Taguchi method of experimental design was applied to determine the optimal parameters of LDW. Results showed that increasing the power led to an increase in the isolation line width and decrease in the surface roughness of the PC within the line. The increase in the pulse repetition rate and defocusing distance caused a decrease in the isolation line width. The optimal parameters were found to be A2B3C3, consisting of power of 5 W, pulse repetition rate of 100 kHz, and defocusing distance of +3 mm. Under these parameters, we obtained an isolation line width of 48.4 μm, and a surface roughness of Ra 38 nm of the PC within the isolation line. We confirmed that the ITO films separated by the isolation lines attained electrical isolation.

## 1. Introduction

Indium tin oxide (ITO) thin films are widely utilized in the flat panel display industry. On account of their excellent light penetration, conductivity, and infrared light reflectivity, ITO films have been considered for use in conductor applications in solar cells, touch panels, electronic ink panels [1], liquid crystal displays [2], flat panel displays [3], and other transparent electrodes [4]. The traditional electrode patterning technique involves photolithography and chemical wet etching processes for the fabrication of film electrode patterns on a substrate. Standard manufacturing processes of film electrodes include sequential photoresist coating, soft baking, exposure, lithography, hard baking, wet etching, and photoresist stripping [5]. The whole process is complex and requires expensive semiconductor equipment. In addition, the wet etching process is generally accompanied by numerous issues, for instance, the need to treat chemical waste, under-cut effects, swelling, significant expense, and substrate [6]. To reduce the large amount of equipment investment required in semiconductor lithography technology and reduce chemical hazards to the environment, the laser direct-write (LDW) technique has been applied for the formation of circuit patterns on ITO films. Lasers enable the confinement of energy in a limited small space, resulting in a highly controllable processing zone with high preciseness of fabrication. Computer-aided laser scanning realizes high-speed manufacturing of arbitrary shapes [7].

Yavas et al. discussed the absorption rate of ITO films using a Q-switch Nd:YLF laser and flash lamp-pumped Nd:YAG laser to ablate the ITO films [8,9,10]. In the study by Ghandour et al., an excimer laser of energy density 350 mJ/cm^2^ was used to produce an isolation line with a width of 11 μm, and the 150 nm ITO film was completely removed to achieve an insulating state [11]. Park et al. utilized an ultrafast laser to remove an ITO film deposited on a glass plate and they found that the ablation threshold of the ITO film was lower than that of the glass [12]. This suggests the selective ablation of the ITO film without damaging the glass substrate [12]. In Tanaka’s experiments, both nanosecond lasers (Nd:YLF, 1047 nm, 10 kHz) and femtosecond lasers (Ti:Sapphire, 800 nm, 1 kHz) were applied to perform the cutting of ITO film on a glass substrate. It was demonstrated that the damage to the edge of the ITO groove, due to the thermal effect, was significantly decreased when cut by the femtosecond laser [13]. Chen et al. used laser beam shaping technology for scribing ITO films. After laser patterning, the morphology of the patterned ITO film was uniform, and no defects caused by the laser on the plastic and glass substrate was observed [14]. Polycarbonate (PC) has a good impact strength, light weight, transparency to visible light, and can be fabricated into complex shapes [15,16,17,18,19]. ITO/PC is mainly used as the bottom electrode in the resistive touch panel structure to replace ITO/glass in order to meet the requirements of electronic products with light, thin, short, small, and flexible properties. However, few studies have investigated the use of LDW on ITO films on PC substrates. In this study, a ITO/PC was patterned using LDW. The effects of the LDW parameters (power, pulse repetition rate, and defocusing distance) on the pattern quality (isolation line width, depth, and surface roughness of the PC within the isolation line) were investigated. In addition, we applied the Taguchi technique to design the experiments.

## 2. Experimental Process

### 2.1. Experimental Equipment

The LDW process was carried out using an ytterbium-doped fiber laser (50 W, BRIMO Technology, Hsinchu, Taiwan), emitting a continuous fiber laser of wavelength 1064 ± 5 nm. In this system, the f-theta (f–θ) centering focal point with a central length of 320 mm and a scanning area of 200 mm × 200 mm was utilized. The laser scanning mode used was X–Y galvanometer scanning and the scanning speed was in the scope of 5–5000 mm/s. The pulse repetition rate was from 70 to 500 Hz.

### 2.2. Material and Experimental Setup

The PC samples with a thickness of 127 μm were coated by DC magnetron sputtering with a 97 ± 20 nm thick ITO film. ITO/PC samples were cut into 250 mm × 200 mm segments and used in the experiments, after fixing on the surfaces of a stainless-steel plate. The laser powers used were 4, 5, 7.5, and 10 W, laser scanning speed was 500 mm/s, and pulse repetition rate was 70, 85, and 100 kHz. The parameter with defocusing distance +0, +1, and +3 mm above the ITO/PC samples was named as Z0, Z1, and Z3, respectively. After scanning a straight line of length 5 cm, confocal laser scanning microscopy (CLSM; KEYENCE, Osaka, Japan) was applied to observe the surface topography, and measure the width, depth, and surface roughness of the isolation line. The surface morphology of the isolation line was observed by a scanning electron microscopy (SEM, JOEL, Tokyo, Japan). Before SEM observation, a thin Au film was deposited on the sample by sputtering in order to increase the electrical conductivity and prevent the accumulation of electrostatic charge at the sample surface. The sheet resistances of the ITO film between the isolation lines were measured by the four point probe method. The electrodes contacted two ITO/PC surfaces which were separated by an isolation line. The optimal laser parameters were determined using the Taguchi method, and the parameters are listed in Table 1. Finally, the data were analyzed using the Taguchi method.

### 2.3. Taguchi Method

In this study, to design the experiment according to the Taguchi method, the following steps were implemented.

(a) Identify the quality characteristics and process parameters to be evaluated.
(b) Determine the number of levels for the process parameters and possible interactions between the process parameters.
(c) Select the appropriate orthogonal array and assign the process parameters to the orthogonal array.
(d) Conduct the experiments based on the arrangement of the orthogonal array.
(e) Analyze the experimental results utilizing the signal-to-noise (S/N) ratio and statistical analysis of variance [20,21].

In the S/N ratio, signal refers to the real value which is desired, and noise refers to the undesired factors in measured values. There are three fundamental classifications to decide the best consequences of experiments; the smaller-the better-characteristic formulas used in this study are given below:

(1) $$S/N_{i}=-10\log_{10}\left[\frac{1}{n}\sum_{i=1}^{n}y_{i}{}^{2}\right]$$

where *n* is the number of measurements, and *y_i_* is the ith observation. The unit of S/N ratio is dB (decibel).

Table 2 lists the combinations of parameters for nine experiments, arranged as an orthogonal array. The average S/N ratio was calculated for each factor, and the largest S/N ratio for each factor yielded the best experimental results. Each experiment was performed three times. All statistical analysis was performed using Microsoft Excel.

### 2.4. Analysis of Variance (ANOVA)

Analysis of variance (ANOVA) was used to examine all controllable factors influencing the width and depth of the isolation line, and surface roughness of the PC, and to determine the most significant factors affecting the quality. The parameters used in ANOVA were calculated using the following equation [22]:

In this study, the various types of parameters were power, pulse repetition rate, and defocusing distance, and *n* (number of samples in each group) was 3. The total sum of squares (SS_T_) was calculated according to below.

(2) $${\mathrm{SS}}_{T}=\sum_{i=1}^{n}{\left(\eta_{i}-\eta_{m}\right)}^{2}$$

where *η_i_*: S/N was calculated from measured isolation line width; *η_m_* is the average value of the calculated S/N ratios from measured isolation line width. SS_T_ consists of the total sum of squares of each factor. A, B, and C factors represented power, pulse repetition rate, and defocusing distance, respectively.

(SS_T_ = SS_A_ + SS_B_ + SS_C_).

(3) $${\mathrm{SS}}_{A}=\sum_{i=1}^{k_{A}}n_{Ai}{\left(\eta_{Ai}-\eta_{m}\right)}^{2}$$

where, *k_A_* is the number of levels of A factor, *n_Ai_* is the number of experiments of A factor at the *i*-level, *η_Ai_* is the S/N ratio of the A factor at the *i*-level, and *η_m_* is the average S/N ratio.

### 2.5. Target Level of the Isolation Line

The target level of the depth of the isolation line was set higher than 97 nm which is thickness of the ITO film. The roughness of the isolation line should be lower or equal to the average roughness of the PC surface (Ra 39 ± 4 nm). The width of the isolation line we set was as low as possible.

## 3. Results and Discussion

### 3.1. Observations of the Isolation Line

**Figure 1:**
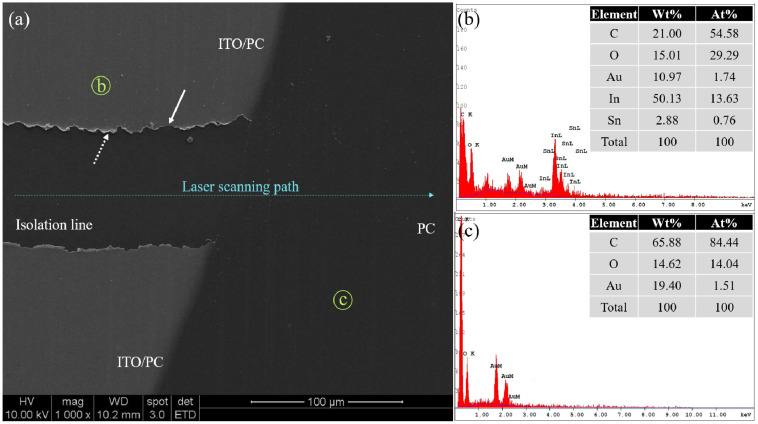
(**a**) Surface morphology of the ITO/PC and PC was treated by laser. (**b**) is EDS spectrum of site b in (**a**). (**c**) is EDS spectrum of site c in (**a**).

In order to understand the effect of the laser on the ITO and PC, laser scanning was performed on the ITO and PC. Figure 1a shows SEM micrograph of the ITO film and PC substrate scanned by LDW. Under the laser radiation, a significant scanning path was observed on the ITO film, suggesting that the ITO film was removed. However, the laser scanning trace was not observed on the PC substrate. Hence, only the ITO film absorbed the laser with wavelength of 1064 nm. In addition, the margin of the ITO film has two characteristics. One is that some areas have sharp edges (indicated by an arrow in Figure 1a) caused by brittle fracture of the film. The other is that some areas show pile-up of melted material as indicated by a dotted arrow in Figure 1a. This implies that some areas of the ITO film solidify quickly and other areas solidify slowly which results in different margin morphologies. The pile-up or shoulder is attributed to the melted material accumulates and solidifies at the edge of the ablation line, which is frequently observed in the laser ablation process [4,23,24].

**Figure 2:**
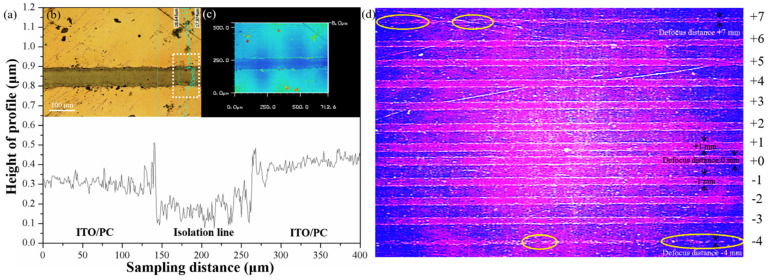
(**a**) Height profile of the isolation line. (**b**) Top view and (**c**) topography image of the ITO/PC with an isolation line. (**d**) The image of the isolation lines abraded by laser with defocusing distance from −4 to +7 mm.

Figure 2a shows the height profile of the isolation line which was measured by the CLSM. The measured region of the isolation line was marked by the white square in the OM image of the top view of the sample (Figure 2b). In Figure 2b, the ITO was ablated by LDW and a consequent groove was observed. The color lines in Figure 2b were the sites where the width, depth, and roughness of the isolation lines were measured. Furthermore, the topography image of the ITO/PC with an isolation line was shown in Figure 2c. Figure 2d shows the image of the isolation line scribed by the LDW while varying the defocusing distance from −4 to +7 mm. Under high defocusing distances (−4 and +7 mm), the isolation line was not continuous and the border of the isolation line was harsh, as indicated by the circles in Figure 2d. It is because the energy density of laser beam is too low to remove the ITO film completely. In the beginning, the heat energy of laser scanning has not been accumulated. After the laser scanning for a certain distance, the heat energy is accumulated due to the overlap of the spots. It results in the center of the lines are clear and the far two ends of the lines are poorly defined. Additionally, at higher defocusing distances (−3, −2, +4, +5, and +6 mm), narrower isolation line widths were observed, but the isolation line widths were not uniform. When the focal position was above the surface of the sample, the laser spot could not focus on the PC substrate, and therefore, the damage of the PC substrate was avoided. Hence, the defocusing distance set in this experiment was required to be 0 to +3 mm.

### 3.2. Effects of the Pulse Repetition Rate on Isolation Line Width and Depth, and Surface Roughness of PC

**Figure 3:**
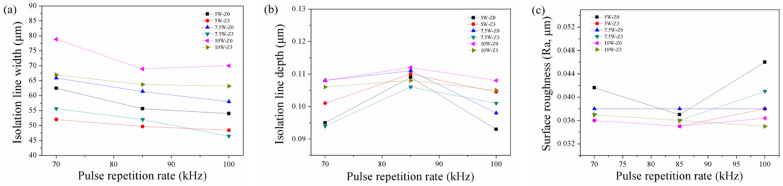
Relationship between the pulse repetition rate and (**a**) isolation line width, (**b**) isolation line depth, (**c**) surface roughness of PC within the isolation line.

Figure 3a shows that the isolation line width decreased with the pulse repetition rate, under the condition of using 5, 7.5, and 10 W power, with defocusing distances of 0, and +3. This result is consistent with that reported by Chen et al., who made patterns on ITO/glass by using an Nd: YAG laser [6]. As the pulse repetition rate increased, the single-shot pulse energy decreased, and the isolation line width decreased. As seen in Figure 3b, when the frequency was increased from 70 to 100 kHz, the depth of the isolation line first increased and then decreased. When the frequency was 70 kHz, the ITO film in the overlapping scan area was removed owing to the high energy of the single pulse; but the PC substrate had thermally expanded, thus resulting in a shallow depth of the isolation line. Moreover, Figure 3c shows that the lowest surface roughness was obtained at a pulse repetition rate of 85 kHz.

**Figure 4:**
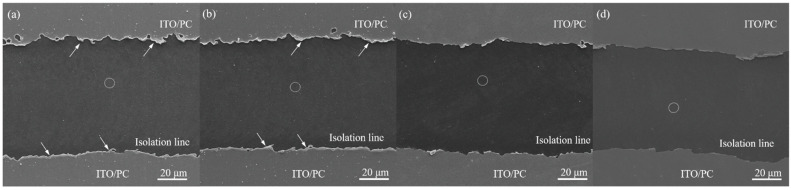
Surface morphologies of the isolation line ablated by 5 W with different pulse repetition rates: (**a**) 70, (**b**) 80, (**c**) 90, and (**d**) 100 kHz.

Figure 4 shows the surface morphology of the isolation line ablated by 5 W powder with different pulse repetition rates. The dark area refers to the isolation line, while the bright area is the ITO/PC substrate. The composition of the isolation line detected by EDS is shown in Table 3. The detected area was marked by a dotted circle in Figure 4. Table 3 indicated that very low amounts of IN and Sn were detected on the isolation line, which may come from small chips absorbed on the surface. In addition, the isolation lines created by low repetition rates (70 and 80 kHz) have a rougher edge, as indicated by arrows in Figure 4a,b, in comparison with those made by high repetition rates (90 and 100 kHz). The low repetition rate means that the number of laser pulses is low, but the single-shot pulse presents high energy. The heat energy caused by the ITO was evaporated and melted. The melted materials would accumulate in the edge of the line, as indicated by arrows in Figure 4a,b, when 70 and 80 kHz repetition rates were applied. However, under the 100 kHz repetition rate, the single-shot pulse with low energy resulted in the smooth line edges (Figure 4d) and a low depth of line. According to the measured results from Tseng’s study, the ITO film (30 nm) on PC has sheet resistance of 433 Ω/Sq [4]. Furthermore, Eshaghi et al. indicated that the ITO film with 200, 300, and 400 nm has 87, 22, and 10 Ω/Sq, respectively [25]. These results implied that the sheet resistance of the ITO film increased with decreasing film thickness. The 97 nm thick ITO film on the ITO/PC used in this study has an average sheet resistance of 41.3 ± 11.1 Ω/Sq. The sheet resistances of the ITO films across the isolation lines prepared by 5 W with 70, 80, 90, and 100 kHz (Figure 4) were measured by the four point probe method. All of them have sheet resistances higher than 9.9 MΩ/Sq, suggesting that they attained the electrical isolation purpose.

### 3.3. Effects of the Defocusing Distance on Isolation Line Width and Depth, and Surface Roughness of PC

**Figure 5:**
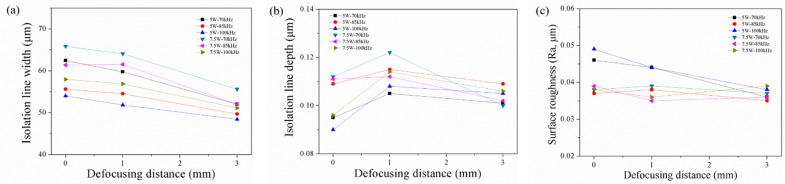
Relationship between the defocusing distance and (**a**) isolation line width, (**b**) isolation line depth, and (**c**) surface roughness of PC within the isolation line.

Figure 5a shows that the isolation line width decreased with the defocusing distance, under the conditions of 5 and 7.5 W power, at pulse repetition rates of 70, 85, and 100 kHz. Larger defocusing distances caused bigger spot sizes, with lower power density in the middle of the spot and, hence, resulted in smaller isolation line widths. Figure 5b shows that the isolation line depth increased first and then slightly decreased, with increasing defocusing distance. Figure 5c shows that the surface roughness of the groove surface decreased with increasing defocusing distance.

### 3.4. Effects of Laser Power on the Isolation Line Width and Depth, and Surface Roughness of PC

**Figure 6:**
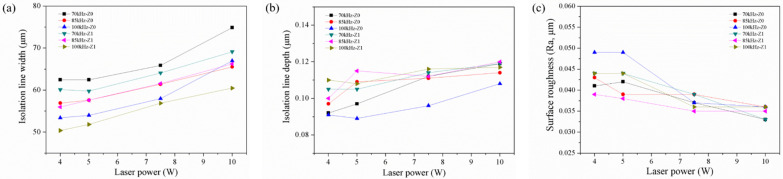
Relationship between the laser power and (**a**) isolation line width, (**b**) isolation line depth, and (**c**) surface roughness of PC within the isolation line.

We attempted to apply 2.5 W laser power with 70, 85, and 100 kHz repetition rate on the ITO/PC. The widths of the isolation lines ablated by 2.5 W were not uniform. Furthermore, using 2.5 W laser power with 100 kHz cannot form a continuous line on the ITO/PC. This implied that the energy density was too low to reach the ITO removal threshold. Accordingly, we chose a level of power higher than 2 W (i.e., 4, 5, 7.5, and 10 W) for further study. Figure 6a–c show the width, depth and surface roughness of the isolation line as a function of the laser power, respectively. In Figure 6a, under the condition of using 70, 85, and 100 kHz pulse repetition rate, with a defocusing distance of 0 and 1 mm, the isolation line width increased with a rise in the laser power, and this result was consistent with that of a previous study [26]. The increased laser power also caused a deeper isolation line depth (Figure 6b), suggesting that the PC below the ITO film was damaged with too high a laser power. This result was consistent with that of a previous study [27]. In Figure 6c, the surface roughness of the isolation line surface decreased with increasing laser power, which could be attributed to the fact that high-energy input is absorbed by the ITO and the heat energy transfers to underneath the PC. This resulted in the PC being able to maintain the melted state for a relatively long time, which allowed the flattening of the PC surface.

### 3.5. Signal-to-Noise (S/N) Ratio Analysis

**Figure 7:**
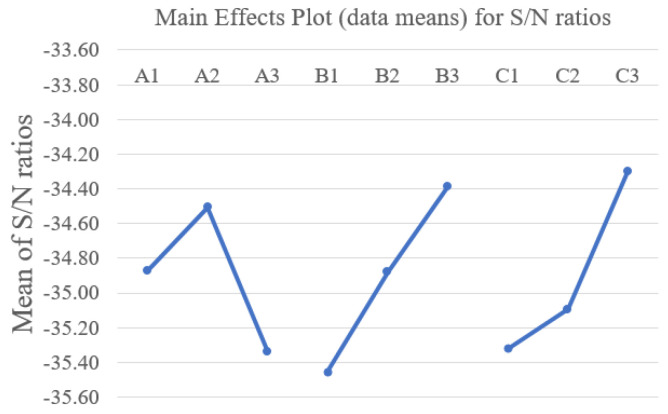
Main effect plot for S/N ratios of the minimum isolation line width.

The isolation line width and surface roughness of the PC within the isolation line of the L9 experimental design are listed in Table 4. Additionally, to determine the relative effects of the factors, the S/N ratio was calculated for the isolation line width, as shown in Table 4. The statistical reliability of the obtained results was calculated. According to Equation (2), the total sum of squares (SS_T_) was calculated; and SS_T_ specified the total variability of the S/N ratio. Table 5 lists the SS_T_ obtained from each factor. The ANOVA results in Table 5 indicate the influence of process parameters on the isolation-width: power with a contribution of 23.1%, pulse repetition rate with a contribution of 37.8%, and defocusing distance with a contribution of 37.7%. The optimal level for obtaining a small isolation line width was chosen to be A2B3C3, corresponding to a power of 5 W, pulse repetition rate of 100 kHz, and defocusing distance of +3 mm. The main effect plot for S/N ratios of the minimum isolation line width was shown in Figure 7. Under the A2B3C3 parameters, we obtained an isolation line width of 48.4 μm, and a surface roughness of Ra 38 nm of the PC within the isolation line. The sheet resistance measurement shows that the isolation lines cause the ITO films nearby the lines to have high electrical isolation properties with a sheet resistance that is higher than 9.9 MΩ/Sq.

## 4. Conclusions

In this study, isolation-lines on an ITO/PC plate were formed using the LDW technique. The effects of the LDW parameters (power, pulse repetition rate, and defocusing distance) on the isolation line width was investigated. The results showed that the isolation line width increased with increasing power and decreased with the increasing pulse repetition rate and defocusing distance. Using the Taguchi method, the LDW parameters of the ITO/PC isolation line were optimized. To obtain a small isolation line width, the optimal levels were chosen to be A2B3C3, corresponding to a power of 5 W, pulse repetition rate of 100 kHz, and defocusing distance of +3 mm. An isolation line width of 48.4 μm, and a surface roughness of Ra 38 nm of the PC within the isolation line was obtained under the optimal parameters.

## Figures and Tables

**Table 1:** Parameters of experiments and levels.

Parameter	Level 1	Level 2	Level 3
Power (W)	4	5	7.5
Pulse repetition rate (kHz)	70	85	100
Defocusing distance (mm)	0	+1	+3

**Table 2:** Tabulation of levels according to L9 orthogonal array.

Experiment No.	Power (W)	Pulse Repetition Rate (kHz)	Defocusing Distance (mm)
1	4	70	0
2	4	85	+1
3	4	100	+3
4	5	70	+1
5	5	85	+3
6	5	100	0
7	7.5	70	+3
8	7.5	85	0
9	7.5	100	+1

**Table 3:** Element content of the isolation line area in the site marked by circle in Figure 4 by EDS.

Element	At %			
	a	b	c	d
C	85.71	86.51	86.46	88.05
O	12.22	11.32	11.82	9.93
Au	1.48	1.75	1.31	1.59
In	0.28	0.25	0.33	0.44
Sn	0.31	0.17	0.07	0
Total	100	100	100	100

**Table 4:** The isolation line width with calculated S/N ratios.

Parameter	Isolation Line Width	
Experiment No	Average Width (μm)	S/N
1	62.6	−35.94
2	55.7	−34.93
3	48.7	−33.76
4	57.8	−35.25
5	49.9	−33.97
6	51.8	−34.29
7	57.3	−35.18
8	61.2	−35.74
9	56.9	−35.11
Average S/N ratio	-	−34.91

**Table 5:** For isolation line width, average S/N ratios of factors and levels, ANOVA table according to Taguchi L9 experiment design and comparison of optimum levels and values obtained by Taguchi method. (A) Degree of freedom, (B) Sum of squares (SS_T_), (C) Average of sum of squares (variance), (D) Effect of factors (%), (E) Optimum Levels, (F) Optimum Values (theoretical).

Factors	1st Level	2nd Level	3rd Level	A	B	C	D (%)	E	F
Power (W)	−34.87	−34.51	−35.34	2	1.05	0.53	23.1	2	5
Pulse repetition rate (kHz)	−35.46	−34.88	−34.39	2	1.73	0.86	37.8	3	100
Defocusing distance (mm)	−35.32	−35.10	−34.30	2	1.72	0.86	37.7	3	+3
Error	-	-	-	2	0.06	0.03	1.4	-	-
Total	-	-	-	8	4.56		100	-	-

## Data Availability

Data sharing is not applicable.
